# Application of Microfluidic Methodology for the Analysis of DNA

**DOI:** 10.3390/mi9010018

**Published:** 2018-01-02

**Authors:** Kirsty Shaw, Yi Heng Nai, Stephen Haswell

**Affiliations:** 1 Faculty of Science and Engineering, Manchester Metropolitan University, Chester Street, Manchester, M1 5GD, UK; 2 Centre for Regional and Rural Futures, Deakin University, Waurn Ponds, VIC 3216, Australia; ryan.nai@deakin.edu.au (Y.H.N.); s.haswell@deakin.edu.au (S.H.)

Over the past 20 years, many of the developments and potential applications of microfluidic methodology have incorporated nucleic acid processes which have, in their own right, undergone a number of innovative changes. We have seen, for example: the materials used for fabrication range from glass to polymers and paper; a wide variety of channel and cavity designs described incorporating both thermal cycling and isothermal temperature control; hybridisation, electrophoretic separations and detection based on both optical and electrochemical methodologies. The various functional combinations of microfluidic-based devices and nucleic acid processing have seen a number of so called ‘on’ and ‘off’ chip processes reported, ranging from single chamber amplification to full extraction, amplification, separation and detection on-chip. It is of course no coincidence that the development of microfluidic technology and nucleic acid processing have advanced hand in hand, primarily in biomedical and forensic areas. The pull for rapid, low cost, at point of need applications, as offered by microfluidic methodology, has acted as the driving force in the innovation of such technology.

This Special Issue will highlight some of the key research areas in the field that support the current state of the art of nucleic acid (RNA and DNA) processing in microfluidic devices. The focus of this Special Issue will however be directed more to the methodology used rather than the relevance of the application described. To this end, the Special Issue will feature seven papers which cover device fabrication, sample introduction, sample preparation and control systems.

The mini review by Birch and Landers [1], not only considers the relevance of a range of electrode type materials for on-chip capillary electrophoretic (CE) applications but also offers an excellent introduction to both DNA and electrokinetic processing in microfluidic devices, well worth a read. The simplification of fabrication methodology to produce fairly complex microfluidic devices has benefited greatly in recent years from the introduction of low cost equipment, such as printers and laminators, coupled with new materials, in particular polymer films and adhesives. This is eloquently presented in the paper by Birch et al. [2] which describes the so called “print, cut, laminate” method for fabricating microfluidic structures in thin film polymers and electrodes that only a few years ago would have required specialized, expensive fabrication equipment and facilities. The paper describes the role a heat-sensitive adhesive plays in the fabrication process to produce fluidic structures which when coupled to a cyclic olefin copolymer (COC) produces a chip capable of DNA extraction, PCR amplification and electrophoretic separation.

In the often neglected area of sample introduction, the paper by Wimbles et al. [3] describes the extraction of DNA directly from FTA^®^ paper used to collect, lyse and preserve a range of sample types of forensic interest. The samples are able to be added directly to a chamber in the glass microfluidic device on top of pre-loaded PCR reagents which are protected by a waxy layer. Following clean-up of the samples by electrokinetic flow of buffer solutions, the PCR reagents were released by heating, producing a simple integrated system. In a further sample introduction example, the combination of cell lysis and DNA pre-concentration of pathologically important bacteria present at very low concentrations in natural waters has been described [4]. Once again, the use of DNA electrokinetic mobilisation, and in this instance a nanoporous membrane, demonstrates a creative way to add value to the analytical process, whilst facilitating the introduction of a sample for downstream processing.

Following sample addition, cell lysis is commonly required to release nucleic acids, however, chemical or enzymatic cell lysis methods can have adverse effects on downstream processes in integrated systems. Branch et al. [5] have evaluated a miniature bulk acoustic wave transducer array to overcome these limitations, and demonstrated extraction efficiencies of nearly 70% in just 10 min.

Most microfluidic devices require associated external control systems to provide functions such as heating or movement of reagents/solutions. Non-instrumented nucleic acid amplification tests (NINAAT) offer all the advantages associated with NAATs, such as high sensitivity and specificity, but without the need for any external instrumentation. Self-regulated and thermostat-controlled resistive heating elements were evaluated by Pardy, Rang and Tulp [6] for their ability to support NINAAT systems. A thermostat-regulated system was found to produce optimal results and was successfully demonstrated for loop-mediated isothermal amplification. An alternative isothermal amplification methodology was shown by Kalsi et al. [7] who incorporated recombinase polymerase amplification into a programmable digital microfluidics platform. Automated dispensing, processing and mixing protocols enabled the simultaneous detection of three antibiotic resistance genes, approximately twice as fast as a conventional benchtop assay.

The editors hope the collection of papers in this Special Issue gives a glimpse of the importance microfluidic methodology offers for the innovative integration of sample processing and analysis which in turn leads to the realization of the micro Total Analytical System (µTAS). Clearly the development of µTAS methodology is in its infancy but, as it develops, the future prospect of fully integrated measurement capability embedded into processes and systems will shape the role of measurement science in the technology and products of the future.
